# The Feasibility and Validity of a Remote Pulse Oximetry System for Pulmonary Rehabilitation: A Pilot Study

**DOI:** 10.1155/2012/798791

**Published:** 2012-09-24

**Authors:** Jonathan Tang, Allison Mandrusiak, Trevor Russell

**Affiliations:** Division of Physiotherapy, School of Health and Rehabilitation Sciences, The University of Queensland, St Lucia 4072, Brisbane, QLD 4072, Australia

## Abstract

Pulmonary rehabilitation is an effective treatment for people with chronic obstructive pulmonary disease. However, access to these services is limited especially in rural and remote areas. Telerehabilitation has the potential to deliver pulmonary rehabilitation programs to these communities. The aim of this study was threefold: to establish the technical feasibility of transmitting real-time pulse oximetry data, determine the validity of remote measurements compared to conventional face-to-face measures, and evaluate the participants' perception of the usability of the technology. Thirty-seven healthy individuals participated in a single remote pulmonary rehabilitation exercise session, conducted using the eHAB telerehabilitation system. Validity was assessed by comparing the participant's oxygen saturation and heart rate with the data set received at the therapist's remote location. There was an 80% exact agreement between participant and therapist data sets. The mean absolute difference and Bland and Altman's limits of agreement fell within the minimum clinically important difference for both oxygen saturation and heart rate values. Participants found the system easy to use and felt confident that they would be able to use it at home. Remote measurement of pulse oximetry data for a pulmonary rehabilitation exercise session was feasible and valid when compared to conventional face-to-face methods.

## 1. Introduction

Chronic obstructive pulmonary disease (COPD) is a progressive lung disease which is characterized by airway obstruction and lung parenchyma destruction. Patients may experience dyspnoea or shortness of breath, a persistent cough with sputum production, decreased exercise tolerance and decreased quality of life [1, 2]. Globally, COPD is the fourth most common cause of death and is predicted by the World Health Organisation to be a major health concern in the coming decade [3]. In Australia, 1.2 million people are estimated to have moderate-to-severe COPD (GOLD stages II–IV). However, the true number is potentially much higher as COPD is commonly underdiagnosed [4, 5]. The economic burden of COPD is substantial. The total cost of living with COPD (including nonmonetary costs) is estimated to be $83 000 AUD per person each year [6, 7]. 

As the severity of COPD greatly impacts the cost of care, providing early diagnosis and effective management of the individual is an essential component in reducing its economic impact [5, 8–10]. One form of effective treatment is pulmonary rehabilitation (PR). PR is a multidisciplinary approach which incorporates self-management education with exercise training, psychosocial and nutritional support [11, 12]. It is a fundamental part of managing people with COPD and has been shown to be effective in improving exercise tolerance, reducing dyspnoea, improving, quality of life and reducing the severity of acute exacerbations [1, 11–17].

Although PR has been shown to be effective in managing symptoms and reducing hospital admissions, poor participation and adherence are a current problem among outpatient programs [18]. Factors include difficulty travelling to the program's location [19–22], inconvenience of hospital attendance [23], or difficulty accessing programs [24].

Accessing programs is especially problematic for those who live in rural and remote areas [25]. Australia is one of the least densely populated nations (2.7 people per square kilometre) with up to 11.8% of the population residing in outer regional or remote locations [26]. Rasekaba et al. [25] conducted a study in Kyabram (rural Victoria) to assess the efficacy of their PR program and noted that patients who lived outside the town centre were more likely to withdraw from PR as the program was conducted within the town hospital. This finding is also supported by De Angelis and colleagues [27] who found that transport, travel distance, and related costs were the main reason for nonattendance of a cardiac rehabilitation program. 

Difficulty in accessing outpatient programs has led to the development of home-based programs [28]. It is well established that home-based PR programs are safe and effective [13, 29–31] and are recommended by many international guidelines and reviews [2, 11, 12, 17]. Given the effectiveness of independent home-based PR programs one could ask what additional benefits supervised remote-based programs would provide. The main advantage of supervised remote-based programs is the ability to minimise anxiety in both patient and therapist. This benefit has been shown in remote-based cardiac exercise programs where the use of transtelephonic exercise monitoring (TEM) has been used to relieve patients' and trainers' anxiety during exercise [32–34]. Other potential advantages include accurate exercise prescription and safe progressions. It is anticipated that a person with COPD could exercise to a higher intensity without the risk of undetected adverse events (e.g., exercise-induced oxygen desaturation). 

Telerehabilitation is an emerging technology used to facilitate the delivery of rehabilitation services at a distance by combining telecommunication and electronic transmission of health information [35]. It has the potential to provide equitable access for people living in rural or remote areas where access to health service is limited [36]. For telerehabilitation to be successful, however, there must be accurate assessment and outcome measurement tools available to therapists [37, 38]. In PR, one essential tool for management of patients is the pulse oximeter [17]. The pulse oximeter is a noninvasive device which continuously monitors a person's peripheral blood oxygen saturation (SpO_2_) and heart rate (HR) [39]. It is used clinically to measure a person's exercise intensity and warn of exercise-induced desaturation [17].

The concept of remote monitoring of physiological data including SpO_2_ and HR for exercise rehabilitation purposes has received attention in the literature. As early as the mid-1980s, physicians were successful at remotely monitoring a patient's electrocardiogram (ECG) through the analogue telephone network during a home-exercise program [40, 41]. However, the majority of electronic healthcare literature on PR has focused on telemonitoring systems [42–46] or consultations services [36, 47]. There has been little research using e-health to deliver real-time, live supervision of patients for treatment purposes.

This study aimed to develop and verify the use of a remote pulse oximetry system on healthy individuals. Specifically, it aimed to establish the technical feasibility of transmitting pulse oximetry data remotely, to determine the validity of remote measurements when compared to conventional face to face measurements, and to evaluate the participants' perception on the usability of the technology. It was hypothesised that remote pulse oximetry would not only be technically feasible but would also be valid when compared to conventional units and would be easy for participants to use. A favourable result will enable the development of remote pulmonary rehabilitation programs for people living in rural or remote areas.

## 2. Materials and Methods

This study was reviewed and granted ethical clearance from the appropriate Medical Research Ethics Committee at The University of Queensland.

### 2.1. Participants

Thirty-seven healthy individuals were recruited to participate in a single remote cardiopulmonary exercise session. The following exclusion criteria were applied: cardiovascular, respiratory, metabolic, or orthopaedic conditions that would prevent participation in an exercise program, chronic infectious diseases, pregnancy, and mental or physical impairment. 

### 2.2. Equipment

**Figure 1:**
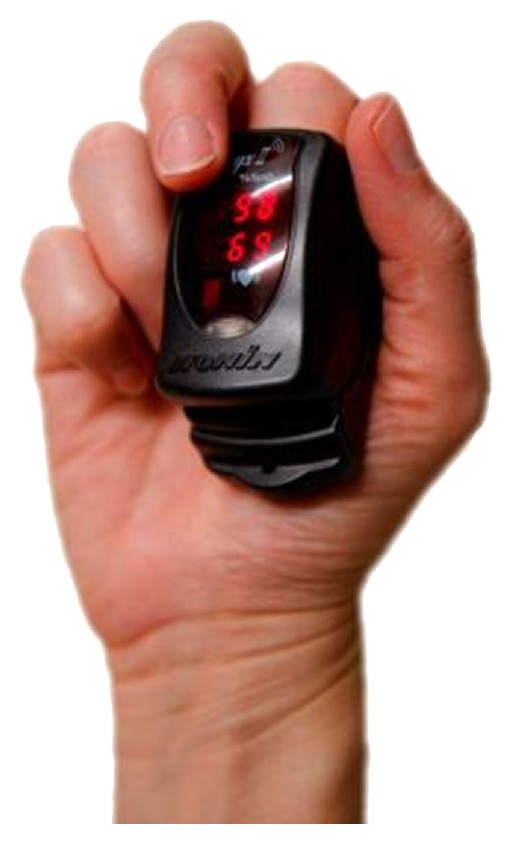
Application of the wireless Bluetooth pulse oximeter module (Onyx II 9560, Nonin Medical Inc., Plymouth, MN).

The exercise session was conducted using the eHAB telerehabilitation system (v2.0, UniQuest, Brisbane, Australia) which is a computer-based videoconferencing and remote consultation system. Two eHAB devices were used, one with the participant and the other with the therapist. The participant and therapist were located in separate rooms. The eHAB device with the participant was configured to receive data from a Bluetooth pulse oximeter module (Onyx II 9560, Nonin Medical Inc., Plymouth, MN) (Figure 1). This information was transmitted by the participant device at a frequency of one Hertz via an encrypted data channel to an offsite server via an active server page (ASP) web service. The data was subsequently stored in a MySQL database on the server. A therapist device retrieved this data via an ASP web service to produce a graph of the participant's heart rate and SpO_2_ recordings in near real time. For this study the eHAB telerehabilitation system utilised a local Wi-Fi network connection; however, the connection speed was throttled to 128 kbit/s to ensure compatibility with the 3G/HSDPA mobile network available in remote areas of Australia. 

### 2.3. Procedure

After written consent was obtained, participants were taken to a room with the eHAB device, remote pulse oximeter module, headset, and treadmill. They were asked to remove any nail polish from their index finger on the nondominant hand or jewellery which may disrupt accurate recordings. Participants were given an information sheet with instructions on how to affix the pulse oximeter module to the index finger of their nondominant hand and how to establish a connection with a therapist in another room via the eHAB device. No other verbal instructions were given. 

Once a videoconference was established, the therapist guided the participant through an exercise session. Each participant performed a 20-minute cardiovascular exercise session on a motorised treadmill. The Modified Borg Score (MBS) [48] was used to assess the participant's exercise intensity. As per current COPD exercise protocols [17], participants were asked not to exercise above an MBS of 5. The session began with a warm-up phase (MBS 2/10) for 5 minutes before gradually progressing to a moderate/somewhat severe intensity (MBS 4-5/10). The last 5 minutes consisted of a cool-down phase (MBS 2/10). If any adverse signs were observed, the exercise session would be ceased immediately. Interrupted sessions due to technical difficulties were noted, and those participants were asked to repeat the exercise session at a later date. 

At the conclusion of the exercise session, participants completed a questionnaire about their experience with the remote pulse oximeter module and eHAB device by marking on a 100 mm Visual Analogue Scale (VAS) and providing any open-ended comments. 

### 2.4. Outcome Measures

During each session, the participant eHAB device logged heart rate (HR) and SpO_2_ values from the pulse oximeter and time stamped the data. This data was saved into a database stored locally on the computer (Dataset #1). The participant device also transmitted the data to the remote therapist device where another time stamp was applied to enable the calculation of transmission latency. This data was saved into a database stored locally on the therapist device (Dataset #2). The mean data transmission rate was determined by measuring the bandwidth requirement (kbit/s). The satisfaction questionnaire rated the comfort, quality, and usability of the eHAB device and remote pulse oximetry module.

### 2.5. Data Analysis

The validity of remote pulse oximetry was assessed by comparing the remote measurements (therapist data) with conventional face-to-face measurements (participant data). Three methods of comparison were used for this purpose. These were percentage agreement, mean absolute difference (MAD), and the Bland and Altman's limits of agreement method [49, 50]. Percentage agreement was calculated as the proportion of data successfully transferred between participant and therapist. The MAD related to the absolute magnitude of errors. The limits of agreement method enabled the quantification of the agreement between the two data sets in terms of the clinical significance of the transmission error. Specifically, it gave the upper and lower bounds which show the 95% confidence interval for the difference between the remote and conventional measurements (i.e., mean ± 1.96 SD). When applying the limits of agreement method comparing the two datasets, the average of 10 second intervals of data was used. 

In terms of clinical significance, the minimum clinically important difference (MCID) was used as a threshold value to represent meaningful and worthwhile change [51]. Remote measurements were classified as valid if the measurement error was lower than the MCID. The commonly accepted MCID for SpO_2_ is ±4 percentage points [52]. The MCID for HR, however, varies depending on the individual's exercise tolerance. For the purpose of this study, the manufacturer's device accuracy of ±3 beats per minute (bpm) was used as the MCID, which was a conservative estimate [53].

Responses from the questionnaire were examined descriptively due to the small sample size. Data was analysed using Statistical Package for the Social Sciences (v17.0 SPSS Inc., Chicago, IL). A *P* value of <0.05 was considered to be significant, where applicable.

## 3. Results

Thirty-seven healthy individuals (14 males and 23 females) completed a total of 15 exercise hours. Participants had a mean age of 30 years and standard deviation of 15 years (range: 18 to 62). There were 34 successfully completed sessions on first attempt confirming the feasibility of the method, while three had to be repeated due to technical difficulties with the Bluetooth or WiFi connection. The mean (SD) transmission latency of the pulse oximetry data (not the videoconference) between the computers was 2.94 (0.59) seconds. The mean (SD) bandwidth usage for data transmitted was 6.5 (1.0) kbit/s.

### 3.1. Validity

There was 80% exact agreement of data between the participant and therapist device. Of the remaining data, 9.0% was found to be omitted from the therapist device, while 11% was found to consist of duplicated numbers. The MAD and limits of agreement between participant and therapist data are shown in Table 1. 

### 3.2. Questionnaires

**Figure 2:**
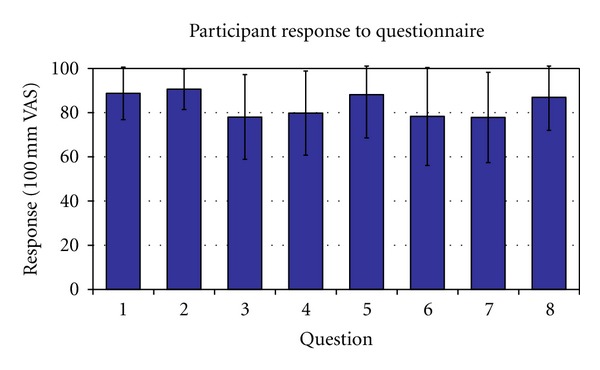
Participant Satisfaction Questionnaire (error bars indicate mean ± 1 standard deviation). High scores indicate a more favourable response than low scores. Questions: (1) how easy was the finger device and headset to operate?; (2) how easy was the device to attach to your finger?; (3) how comfortable was the device during the exercise program?; (4) could you see the physiotherapist clearly during the exercise session?; (5) could you hear everything that was being said?; (6) how confident are you in using technology in general?; (7) how interested are you in learning about new technology?; (8) would you feel confident using this device at home?

Overall, participants rated their experience with the remote pulse oximeter and eHAB device highly. The mean response to each of the eight questionnaire items is shown in Figure 2. Positive written comments included the following: “I would be comfortable using [this device] at home” (*n* = 4) and “[the eHAB was] simple and easy to use” (*n* = 2). Negative comments included worried [remote pulse oximeter] could dislodge during vigorous exercise (*n* = 5), remote pulse oximeter was uncomfortable (*n* = 4), and audio or video quality was “jumpy” (*n* = 3).

## 4. Discussion

Pulmonary rehabilitation has been proven effective in the management of COPD. However, inconvenience, distance, or availability of PR programs can lead to lack of participation and poor compliance. Telerehabilitation is an emerging technology which can potentially address these problems. This study demonstrated the feasibility of a remote pulse oximeter for use during a PR program. Remote measurements of SpO_2_ and HR were valid when compared to conventional face-to-face measurements. These findings provide confidence for the development of a remote PR program. 

In our study, there was an unexpectedly high mean rate of data omission (9.0%) and duplication (11%) between participant and therapist data. Although these error rates appear comparatively high, they do not necessarily indicate a failure in the data transmission. Rather, the logging of data points by the participant device appears to not be perfectly synchronized with data retrieval from the therapist device. This may be a function of the variable delays that can be present with web service calls which were used in this study to transmit data [54]. Asynchrony in web service responses may have resulted in either repetition of data or omission of single values. Moreover, a visual inspection of the data confirms a random distribution of error throughout the data file. Despite this asynchrony, the magnitude of errors was relatively small. The MAD analysis revealed a small difference (SpO_2_ 0.04%; HR 0.21 bpm) in comparison to the MCID of ±4% for SpO_2_ and ±3 bpm for HR. This gives confidence in the validity of remote measurements. 

Similarly, the limits of agreement demonstrated a small difference in comparison to the MCID for SpO_2_ and HR. The upper and lower bounds for SpO_2_ were −0.67 to 0.67% and for HR were −2.90 to 2.89 bpm. Both are within the MCID, and this provides further evidence for the validity of remote measurements. Unlike MAD, which analyses the actual difference, the limits of agreement give a conservative estimation of difference through a 95% confidence interval. Clinically, it means 95% of the time of the difference due to measurement error will be within the relevant limits for SpO_2_ and HR. The MAD and limits of agreement values show that remote measurements are sufficiently accurate and valid for real-time monitoring purposes.

Overall, as indicated by questionnaire responses, participants found the remote pulse oximeter easy to use (mean VAS 89/100) and felt confident that they would be able to use it at home (mean VAS 87/100). This is similar to literature which has found the eHAB device user friendly [55–57]. However, some participants (*n* = 4) stated that the remote pulse oximeter was uncomfortable. Unlike a conventional pulse oximeter, the remote module has the sensor, display and battery unit all incorporated on the fingertip (see Figure 1). This creates additional weight compared to a conventional sensor, and it could be a contributing factor to the feeling of instability experience by these participants. Future studies should compare the comfort with other remote pulse oximeters that have the display and battery unit located at the wrist. Furthermore, this needs to be investigated in the COPD population as they may be more accustomed to pulse oximetry than healthy adults. Five participants commented that they were worried the remote pulse oximeter could dislodge during vigorous exercise. This could be a result of the weight of the device and high intensity of exercise, where most participants needed to jog or run in order to reach an MBS of 5/10. 

Adequate video and audio quality is important for patient confidence. Problems with video and audio quality have been identified as key issues in telehealth [58–60]. During testing, there were occasional disruptions in audio and video transmissions during the exercise session, and on three occasions, the videoconference unexpectedly exited due to connectivity issues on the WiFi network, requiring the call to be reestablished. Additionally, three participants commented on “jumpy” images during the video conference. The low frame rate is likely due to the low bandwidth available (128 kbit/s) on the network connection [61]. However, it should be noted that the remote pulse oximeter only uses a very small bandwidth (6.5 kbit/s) for data transmission and, therefore, is unlikely to have attributed to the reduced video/audio quality. 

There were several limitations of this study (1) Our sample size was small (*n* = 37) and consisted primarily of young, healthy university students (mean age 30 years). Our healthy participants did not show any clinical changes (e.g., exercise-induced desaturation), and this may have affected the magnitude of error in MAD and limits of agreement. Furthermore, the young population may be more confident to use or learn about new technology, and this may have therefore influenced the result of the satisfaction questionnaire. (2) This study was conducted within the university clinic. Although the participant and therapist were in separate rooms, they were in close proximity to each other, with the therapist on call if any difficulties occurred. This support may have also affected participant's satisfaction ratings. (3) A simulated 3G network was used to transmit data. Whist the eHAB system was bandwidth throttled to ensure compatibility with the 3G/HSDPA mobile network, other factors such as mobile network coverage, signal strength, and speed were not assessed, and this may have affected the performance and the latency of transmission. 

Future studies should include a trial of the remote pulse oximetry system in the home environment with a COPD population on the 3G/HSDPA mobile telephone network. A large sample size is required to more rigorously assess the validity and reliability of data transmissions. Furthermore, it has been shown that patients demonstrate more positive views of telecare encounters than their healthcare providers [62]. A therapist questionnaire is therefore required to assess their satisfaction and confidence in safely conducting a remote exercise session. 

## 5. Conclusion

Our pilot study demonstrated that remote pulse oximetry was feasible, and the data transmission method produced valid data at the remote end when compared with conventional face-to-face measures. This provides support for the development of remote pulmonary rehabilitation programs. Although this study was performed on healthy individuals, participants found the eHAB device easy to operate and were confident it could be used at home. Further studies are needed to address the technical feasibility and validity in a home environment and the therapist's confidence in conducting a safe exercise program.

## Figures and Tables

**Table 1:** Statistical comparison between data logged at the participant (local) and therapist (remote) sites.

Parameter	Mean absolute difference (SD)	Limits of agreement	
		Lower bound	Upper bound
Heart Rate (bpm)	0.21 (1.46)	−2.90	2.89
SpO_2_ (%)	0.04 (0.34)	−0.67	0.67
